# Foreign Correspondence

**Published:** 1846-11

**Authors:** 


### THE WESTERN JOURNAL.

NEW SERIES.—VOL. VI.—NO. V.

LOUISVILLE, NOVEMBER 1, 1846.

---

### FOREIGN CORRESPONDENCE.

PARIS, Sept. 16th, 1846.

*To the Editors of the Western Journal:*

GENTLEMEN—In the following letter I shall give you such detached items of information as I have had opportunity to acquire during my short residence in this city, trusting that in my future communications I shall be able to observe more system. Among the many distinguished medical men of the French metropolis, Velpeau was one whose lectures I was most anxious to hear. I had the pleasure of listening to his last cliniques at the Charity Hospital, and an outline of one of these I have attempted to give. I like him as a lecturer; his style is good; viz: plain, and unaffected; his matter is excellent, his remarks sufficiently practical and succinct. I promise myself great pleasure and profit from attendance upon his course, which begins in November and continues during ten months. Velpeau is between fifty and fifty-five years of age, of ordinary stature, and of a peculiar expression of face. His hair is beginning to be gray, while his eye-brows are perfectly black, large and heavy, and, hanging over light hazel eyes, a little sunken, give to him this expression. He is at the Hospital of Charity, and is followed, during his visits, by crowds of both French and American students. He begins these at 8 o'clock in the morning and finishes at 9, when his lecture commences, which continues about an hour. My breakfast hour is now half-past 10 o'clock. In the winter, when the days become shorter, the student, who would be regularly at his post, must rise, dress, and breakfast by candle-light; and he who does this for half the year, most assuredly "scorns delights and lives laborious days."

*Hopital de la Charité—M. Velpeau—Erysipelas.*—There are many points worthy of mention both in the theory, nature, and treatment of the disease called Erysipelas. Since the time of Franck physicians have contended, and M. Chomel has been singularly warm in his advocacy of the opinion, that erysipelas of the head is always preceded by engorgement of the submaxillary ganglia. The evidences which they advance in support of this theory do not appear to us to have been seen in their true light. We regard the ganglionic irritation or engorgement as an effect, a consequence, rather than a cause or precedent of erysipelas. There are numerous instances on record where erysipelas has traversed the scalp and reached the face unnoticed, and we think it risking nothing to say that these are the cases where engorgement of the submaxillary ganglia is settled upon as the cause of the disease.

We endea ored to show in a previous lecture, and we shall return again to the subject, when speaking of diseases of the glandular system, that this mode of regarding the gang. engorge. which is observed in erysipelas agrees in every particular with the manner in which engorgements of the ganglia in general are produced.

In our opinion the idea of the pre-existence of ganglionic engorgements in erysipelas is an error arising from this obvious cause, to-wit: that the first stages of the disease pass unobserved and undetected, while in reality the tumefaction of the ganglia follows the cutaneous inflammation. This remarkable circumstance is connected with the treatment of this disease. There is no mode by which it is arrested; every topical remedy has been applied without seeming to diminish its duration or limit its extent. Mercurial ointment, hog's lard, ointment of calomel, sulphate of copper, diluted acid, nitrate of silver, on which a vast deal has been said, and to the consideration of which an

American physician has even devoted a whole volume—blisters, sinapisms, etc., all have been tried, and all have failed.

We have met with but one application which has appeared to us to possess any value in the treatment of erysipelas; that is the ointment of sulphate of iron.

Sulph. iron 8 ä 10 grammes.
Lard 30 ä 40 grammes.

If you prefer it in the form of solution, add 10 grammes of the sulphate of iron to 100 grammes of water. Compresses saturated in this mixture are to be laid upon the skin.

This salt has evidently some action on erysipelas; the parts over the integuments with which it is placed in contact become white and shrivel up; though it does no more towards circumscribing the progress of the disease than any other compound. As a remedy, therefore, it is not entitled to much attention. In many cases the general symptoms of erysipelas will continue unabated and even increase after the entire disappearance of the redness, a fact too clearly pointing to the conclusion that it is a constitutional affection, and hence beyond the reach of all topical applications.

And although we are disposed to believe that the sulphate of iron has been occasionally used with advantage, we would have it distinctly borne in mind, that a general remedy is what we want and what has not yet been discovered.

We proceed to mention a striking result, which seems at first to be rather the effect of caprice. Out of five patients who were admitted into the hospital for erysipelas not one has died, and among fifteen who were seized with the disease while lying in the wards, the enormous proportion of eight have died. This difference of mortality is most surprising, and as easily accounted for.

The five patients who were brought to the hospital laboring under erysipelas were suffering under this disease alone, and notwithstanding we admit that even under such circumstances as these patients sometimes die, the termination of the attack is very generally in recovery. What now are the facts in relation to the fifteen individuals whom erysipelas seized while inmates of the hospital wards? Every one was enduring disease in some shape or other; some had submitted to operations; one had peritonitis; another pneumonitis; and others again were exhausted by suppuration in different parts of the body.

The fate of these patients, therefore, must be ascribed as much to the maladies under which they labored at the time that erysipelas supervened as to that affection itself.

It must be received as a law equally in medicine and surgery, that erysipelas, existing in complication with other diseases, is always a most serious and frequently a fatal disorder—if not so in itself, at any rate on account of the grave concomitants amid which it is developed. In hydrocele M. Velpeau is particularly partial to iodine injections.

When the rumor that Asiatic cholera had made its appearance in London reached Paris, the Academy of Medicine despatched two of its members to that city in order to study the disease. At one of the meetings of the Society of Practical Medicine held soon after, great wonder and astonishment were expressed by various learned gentlemen who were present, that the Academy had been guilty of so great a blunder. They were confident that Paris afforded equally as fine a field for the investigation of cholera as London. Six of the members of the Society had met with cholera this season too clearly developed to be mistaken. Some had had several cases. The symptoms were vomiting, colorless alvine evacuations, cramps, coldness of the extremities, coldness of the whole surface of the body, with the blue tinge of the skin, sunken eyes, and suppression of urine, which latter symptom existed for thirty-six hours in one instance. The treatment pretty generally consisted in opiate draughts, laudanum and starch injections, frictions, sinapisms to the extremities, cataplasms to the abdomen, and very small quantities of fluids to drink for fear of inducing vomiting.

*M. Belhomme,* who introduced the subject, had practised venesection in a case which he was called to. He first ordered the draught and injection, frictions, etc., and in twelve or fifteen hours after, when some amelioration of the symptoms was observed, venesection was performed, and the next day the patient, a woman of fifty-three years old, had entirely recovered.

In the Medical Gazette of Strasbourg it is stated that the cesarian operation was performed with entire success by Dr. Steinbrenner, on the 15th of January last. On the first of March both mother and child were doing well.

The journals from India of the 15th of August speak of the ravages of the cholera. They also state that an English physician, Doctor Ballguer, has discovered a remedy for this terrible disease. Patients to the number of fifteen had been relieved through his instrumentality. The treatment is simple, consisting in the use of a bath of medicated vapor. This scourge has set its foot in Persia. Teheran has been deserted by the king, his ministers, and the entire court. The diplomatic corps is preparing to follow the example. The journals admit that the disease is communicated by the air. They fear that it will pursue the same track that it did in 1832.

A lad, aged 18 years, fell not long since upon his hands from the fifth round of a ladder upon which he was standing, which resulted in the fracture of the first metacarpal bone of the left hand. Crepitation, with shortening of the bone, contraction of the muscles, etc., characterized the case. Three longitudinal compresses, paste-board splints, and bandages sufficed, in the space of a month, to produce re-union of the bone. This is the only case on record where so small a bone has been fractured by such a force. The power which produced it acted as a lever of the second kind. D. W. Y.

